# The role of dendritic cells in immunity against primary herpes simplex virus infections

**DOI:** 10.3389/fmicb.2014.00533

**Published:** 2014-10-21

**Authors:** Sammy Bedoui, Marie Greyer

**Affiliations:** Department of Microbiology and Immunology, Peter Doherty Institute for Infection and Immunity, The University of MelbourneParkville, VIC, Australia

**Keywords:** dendritic cells, T cells, cytokines, viral immunity, CD40

## Abstract

Herpes simplex virus (HSV) is a DNA virus with tropism for infecting skin and mucosal epithelia during the lytic stages of its complex life cycle. The immune system has evolved a multitude of strategies to respond to primary HSV infections. These include rapid innate immune responses largely driven by pattern recognition systems and protective anti-viral immunity. Dendritic cells (DC) represent a versatile and heterogenic group of antigen presenting cells that are important for pathogen recognition at sites of infection and for priming of protective HSV-specific T cells. Here we will review the current knowledge on the role of DCs in the host immune response to primary HSV infection. We will discuss how DCs integrate viral cues into effective innate immune responses, will dissect how HSV infection of DCs interferes with their capacity to migrate from sites of infection to the draining lymph nodes and will outline how migratory DCs can make antigens available to lymph node resident DCs. The role of distinct DC subsets and their relevant contribution to antigen presentation on MHC class I and MHC class II molecules will be detailed in the context of T cell priming in the lymph node and the elicitation of effector function in infected tissues. An improved understanding of the fundamental mechanisms of how DCs recognize HSV, process and present its antigens to naïve and effector T cells will not only assist in the improvement of vaccine-based preventions of this important viral disease, but also serves as a paradigm to resolve basic immunological principles.

## INTRODUCTION

Herpes simplex virus (HSV) type 1 and 2 are α-herpesviruses of the herpesviridae family, representing one of the most frequently encountered infections in humans (Koelle and Corey, 2008). Infections with HSV typically commence as the virus comes into contact with deeper layers of the epithelium that line the skin, the cornea or the mucosal tissues of the anogenital region through small injuries. HSV enters these cells either by fusing directly with the cell membrane or by translocating into the cytosol following endocytic uptake. Infection of individual cells not only gives rise to multiple newly formed virions that can then spread to other cells, but also causes significant damage to the infected cells that historically is referred to as a cytopathic effect. These changes include enlargement and disaggregation of the nucleolus, chromatin damage, destabilization of the cytoskeleton, insertion of viral glycoproteins into organelle membranes and a global decrease in macromolecular synthesis. The resulting death of multiple infected cells leads to the destruction of the epithelial layer of the skin, mucosa, or cornea. In the context of the skin, accumulation of interstitial fluid between the dermo-epidermal junction results in the development of the characteristic blisters associated with cold sores.

Symptoms of primary HSV infection are usually mild and self-limiting. However, infections of individuals with acquired or inherited immune defects can result in life-threatening disease (Sancho-Shimizu et al., 2011). This highlights the paramount importance of immune control of HSV infected cells and tissues. As with many intracellular infections, effective immune control of HSV relies on the activation of cytotoxic lymphocyte responses, among which HSV-specific cytotoxic T cells (CTL) are particularly potent (van Lint et al., 2004; Gebhardt et al., 2013; Shin and Iwasaki, 2013; Zhu et al., 2013). Before HSV-specific CTLs can seek and destroy infected cells, the few circulating naïve, antigen-specific CTLs need to be activated in secondary lymphoid organs by dendritic cells (DC). In the case of murine HSV skin infections this occurs in the local draining lymph node (Bedoui et al., 2009b). With the primary reservoir of viral antigens located in the epithelial layer of the affected organ, migratory DCs capable of delivering antigens from the periphery to the lymph node therefore play a crucial role in the initiation of antiviral cell-mediated immunity (Zhao et al., 2003; Allan et al., 2006; Bedoui et al., 2009b; Lee et al., 2009; Frank et al., 2012).

The aim of this review is to summarize our current knowledge on how DCs located at sites of primary HSV infection respond to the virus, how this interaction impacts on their capacity to migrate to the local draining lymph node and what role different DC subsets play in the initiation of virus-specific T cell responses. Not withstanding the distinct importance of HSV infections progressing into latency (Roizman and Whitley, 2013) and evidence suggesting an interesting role for DC–T cell interactions in the context of neuronal HSV infections (St Leger and Hendricks, 2011), we will focus on the events that take place during the lytic stage of primary, extra-neuronal HSV infections.

## PIVOTAL ROLE FOR DCs IN THE CONTROL OF PRIMARY HSV INFECTIONS

It is now well established that DCs consist of multiple subsets that differ in phenotype and function (Heath and Carbone, 2009; Reizis, 2012; Merad et al., 2013). Non-lymphoid organs contain at least two types of conventional DCs that are characterized by the expression of either CD11b or CD103 (Heath and Carbone, 2013; Malissen et al., 2014). The epidermis harbors an additional DC subset, the Langerhans cells (LC; Kaplan, 2010). It was initially thought that LCs are the only DC subtype expressing the C-type lectin langerin in the skin, but it was more recently appreciated that CD103^+^ DCs also express langerin (Merad et al., 2008). Common to these DC subsets in peripheral, non-lymphoid tissues is their capacity to depart the tissue of origin, enter lymphatics, and migrate to local lymph nodes, which is why these DCs are often referred to as migratory DCs. Apart from migratory DCs entering the lymph nodes via afferent lymphatics, lymphoid organs also contain a separate, locally resident DC population that can be further classified into CD11b^hi^ and CD11b^lo^ subsets. The CD11b^lo^ subset contains CD8^+^ DCs in mice and human blood-dendritic cell antigen (BDCA3)^+^ DCs in humans (Bachem et al., 2010; Crozat et al., 2010a; Shortman and Heath, 2010). An important distinction between resident and migratory DCs is therefore that only migratory DCs collect antigens in non-lymphoid-tissues, such as the skin, while resident DCs are limited to accessing antigens within the particular lymphoid tissue they occupy. Plasmacytoid DCs (pDC) differ substantially from migratory and resident DCs. While their direct contribution to T cell priming during viral infections remains controversial, it is well established that pDCs are very efficient at secreting key antiviral cytokines, such as interferon (IFN)-α/β (Villadangos and Young, 2008). In addition to conventional DCs and pDCs, inflammation can also drive the *in situ* differentiation of CCR2^+^ Ly6C^hi^ monocytes into inflammatory or monocyte-derived DCs (Mo DC) both in the periphery and lymphoid organs (Dominguez and Ardavin, 2010).

## INNATE RECOGNITION OF HSV BY DCs

The early lytic phase of HSV skin infection brings into play the immune system. The viral insult and the resulting tissue damage not only evoke an inflammatory response leading to neutrophil influx (Stock et al., 2014) and release of pro-inflammatory mediators (Eidsmo et al., 2009; Puttur et al., 2010), but also activate epidermal LCs and dermal CD11b^hi^ DCs (dermal DC). Equipped with a broad suite of pattern recognition receptors, DCs are very effective at detecting pathogens and pathogen-associated molecular patterns (PAMP). Several studies have therefore examined the ability of DCs to recognize HSV and assessed how this recognition leads to the secretion of key anti-viral cytokines. For example, it has been demonstrated that *in vitro* exposure of bone marrow-derived DCs (BM DC) to HSV results in the secretion of IL-6 and IL-12 (Sato et al., 2006). Interestingly, this release of IL-6 and IL-12, at least in response to some HSV substrains, appeared to be regulated by the consecutive stimulation of toll-like receptor (TLR) 2 through viral glycoproteins, such as gH/gL or gB (Leoni et al., 2012), and endosomal TLR9 by viral DNA (Sato et al., 2006). HSV recognition by DCs also elicits the release of IFN-α/β which exert potent antiviral activities such as blocking immediate-early HSV gene expression, preventing release of virions from infected cells, and limiting progression of the infection from peripheral tissues to the nervous system (Noisakran and Carr, 2001). Accordingly, stimulation of IFN-α/β secretion is crucial for effective control of HSV infections, as mice lacking the receptor for IFN-α/β are unable to prevent spread of the virus upon primary infection into the central nervous system (Halford et al., 1997; Leib et al., 1999; Conrady et al., 2012). Although pDCs generally represent a major source of IFN-α/β (Villadangos and Young, 2008) and triggering of TLR9 by HSV elicits IFN-α/β secretion from pDCs (Lund et al., 2003), the relative importance of pDCs seems to depend on the route of HSV inoculation. Vaginal infections (Lund et al., 2006), and to a much lesser degree systemic infections (Swiecki et al., 2013), require pDCs for effective HSV control. However, the absence of pDCs has no impact on viral control following cutaneous infections (Swiecki et al., 2013). These reports together with the demonstration that a lack of myeloid differentiation primary response gene 88 (MyD88), which is a crucial downstream adaptor molecule required for TLR2 and TLR9 signaling, had no effect on HSV replication following subcutaneous or corneal infections (Conrady et al., 2012; Swiecki et al., 2013) imply that alternative pattern recognition receptors also play a significant part in translating HSV recognition into IFN-α/β responses. Recent findings suggest that HSV induces IFN-α/β through a cytosolic DNA sensor system that involves the adaptor molecule stimulator of IFN genes (STING; Ishikawa et al., 2009). Although we are only beginning to unravel the upstream sensor molecules that utilize STING to drive IFN-α/β responses, recent *in vitro* evidence indicates that HSV-induced IFN-α/β secretion by BM DCs requires the Asp-Glu-Ala-Asp (DEAD) box helicase DDX41 (Zhang et al., 2011) and/or guanosine monophosphate–adenosine monophosphate synthase (cGAS; Sun et al., 2013; Wu et al., 2013). Gamma-IFN-inducible protein (IFI) 16, and its murine equivalent IFI-p204, also appear to be likely candidates that link HSV recognition to STING-dependent IFN-α/β responses (Unterholzner et al., 2010; Conrady et al., 2012). In conjunction the available data suggests that DCs respond to HSV through membrane-bound pattern recognition receptors, such as TLR2 and TLR9, and via STING-dependent cytosolic recognition pathways. Considering that DCs can be infected with the virus (see below) and that viral replication exposes the cytosol to viral PAMPs, it is tempting to speculate that the activation of cytosolic receptors largely occurs in DCs that are infected with the virus, while engagement of endosomal TLRs results from uptake of virus-containing material by the DCs.

While the above has focused on how distinct pattern recognition receptors contribute to innate responses by DCs upon HSV recognition, emerging evidence suggests that alternative, pattern recognition receptor-independent pathways might also play a role in innate activation during virus infection. For example, it has been demonstrated that exposure of cells to viruses can result in replication-independent disruptions of membrane integrity and alterations of the cytoskeletal architecture that lead to IFN regulatory factor (IRF) 3 activation and IFN-α/β production (Hare and Mossman, 2013). It will be intriguing to determine how such pattern recognition receptor-independent physical alterations that occur in DCs encountering HSV contribute to their innate immune activation.

## MIGRATORY IMPEDIMENT OF HSV-INFECTED DCs

The interaction between HSV and DCs can also result in productive infection of DCs. This has been demonstrated in murine experiments, where LCs, dermal DCs, and CD103^+^ DCs emigrating *ex vivo* from HSV infected skin explants produced HSV-encoded non-structural virus-derived proteins, such as infected cell protein (ICP) 8, that require *de novo* synthesis (Puttur et al., 2010). Consistent with this, several studies using murine BM DCs or human DCs grown from blood monocytes have shown that DCs can be productively infected with HSV *in vitro*. Remarkably, although HSV-infected DCs are present in primary HSV skin lesions (Eidsmo et al., 2009) and migration of DCs to the draining lymph node is dramatically increased in response to the infection (Allan et al., 2006; Bedoui et al., 2009b; Puttur et al., 2010), productively infected DCs could not be detected in lymph nodes draining the site of infection (Eidsmo et al., 2009). Together with transfer studies of *in vitro* infected and control BM DCs showing that infected DCs are significantly impaired in migrating from subcutaneous tissues into the lymph node (Eidsmo et al., 2009), it seems that HSV-infected DCs are excluded from, or are at least impaired, in migrating from infected skin to the lymph node. Although definitive *in vivo* evidence is pending, *in vitro* studies suggest that this lack of infected DCs reaching the lymph nodes could be due to a combination of different effects the virus exerts on infected DCs. Previous studies have shown that *in vitro* HSV-infected human Mo DCs had reduced chemotactic abilities to follow CCL19 gradients and displayed enhanced attachment to fibronectin (Theodoridis et al., 2011). The authors suggested that enhanced fibronectin binding required viral gene transcripts that block cytohesin-1 activity, thereby increasing lymphocyte function-associated factor (LFA) 1 mediated adhesion to extracellular matrix (Theodoridis et al., 2011). In addition to these integrin-mediated mechanisms potentially retaining infected DCs in the skin, other molecules that regulate the departure of DCs from the skin also appear to be manipulated by HSV. Skin-residing LCs express high levels of E-cadherin that tightly connect these to keratinocytes. To depart the skin and enter the lymphatics, LCs not only need to upregulate expression of CCR7 to follow the CCL19/21 gradients present in the lymph, but also require downregulation of E-cadherin to be released from keratinocytes (Jiang et al., 2007). It was reported that although most LCs obtained from HSV-infected skin downregulated E-cadherin, those infected with HSV retained high expression levels of E-cadherin (Puttur et al., 2010). As the retention of surface E-cadherin occurred only in the presence of live but not UV-inactivated virus, the authors concluded that *de novo* viral gene expression rather than uptake of dead virus by the DCs was responsible for this. While these studies did not observe major differences in the upregulation of CCR7 between infected and uninfected LCs in HSV-infected skin explants, *in vitro* experiments revealed that HSV-infected human Mo DCs were impaired in their ability to respond to CCL19 (Salio et al., 1999) and failed to fully upregulate CCR7 (Prechtel et al., 2005).

## APOPTOSIS OF HSV-INFECTED DCs

Impaired migratory behavior does not appear to be the only means that impedes infected DCs from arriving in lymph nodes, as *in vitro* HSV infections revealed that infected DCs also die more rapidly than controls. Although this was initially believed to be due to the cytopathic effects of the virus, it is now appreciated that HSV can cause apoptosis of infected cells (Nguyen and Blaho, 2007). *In vitro* studies revealing that inhibitors of protein synthesis prevented HSV-induced apoptosis have led to the concept that the virus actively initiates apoptotic cascades. Consistent with these studies that were largely conducted in Vero or HeLa cells (Nguyen and Blaho, 2007), *in vitro* HSV infections of human, macaque, and murine Mo DCs or BM DCs also result in caspase-3 activation and significant DC death (Jones et al., 2003; Peretti et al., 2005). Whether HSV-induced DC death resulted from the transcription of viral genes that actively induced apoptosis is not entirely clear as some studies found that UV-inactivation abrogated the ability of HSV to kill infected DCs (Jones et al., 2003), while others reported that UV-inactivated virus was still capable of inducing apoptosis in DCs (Stefanidou et al., 2013). Regardless of the underlying molecular events, it is clear that HSV-infected DCs can be driven into apoptotic cell death following infection.

**FIGURE 1:**
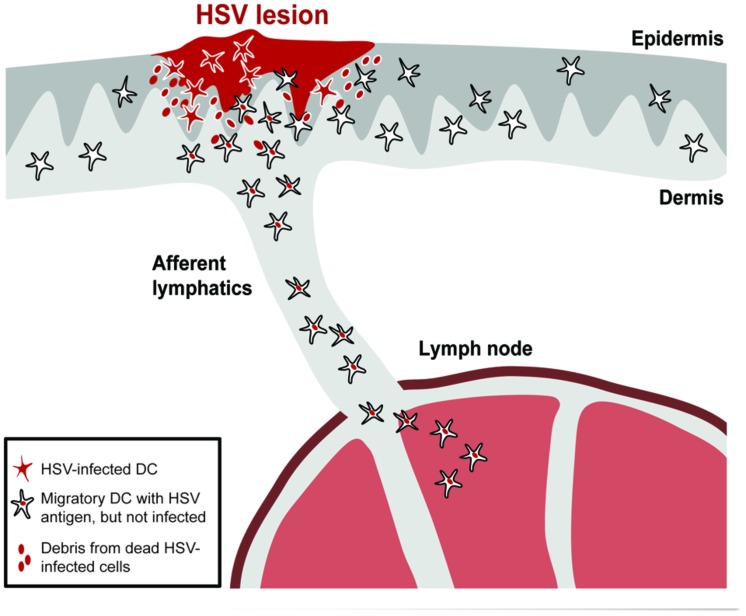
**HSV-infected DCs are retained in the skin and excluded from migrating to the lymph node.** Skin DCs, such as Langerhans cells (LC), dermal DCs, and CD103^+^ DCs, can be productively infected in HSV-infected skin. This leads to their retention in the skin and induction of apoptosis. Debris from dying or dead infected cells will be taken up by uninfected migratory DCs that then depart the skin to transport the antigens to the lymph node via afferent lymphatics.

Overall, a picture emerges in which infected DCs are prevented from migrating from infected tissues to the local draining lymph nodes (**Figure 1**). As uninfected DCs still migrate from infected sites, this phenomenon is most likely related to selective migratory impairments in infected DCs, which on a functional level are associated with sustained retention in the skin, poorer responsiveness to chemokine gradients and induction of apoptosis.

## STIMULATION OF HSV-SPECIFIC CTLs BY DCs IN THE LYMPH NODE

**FIGURE 2:**
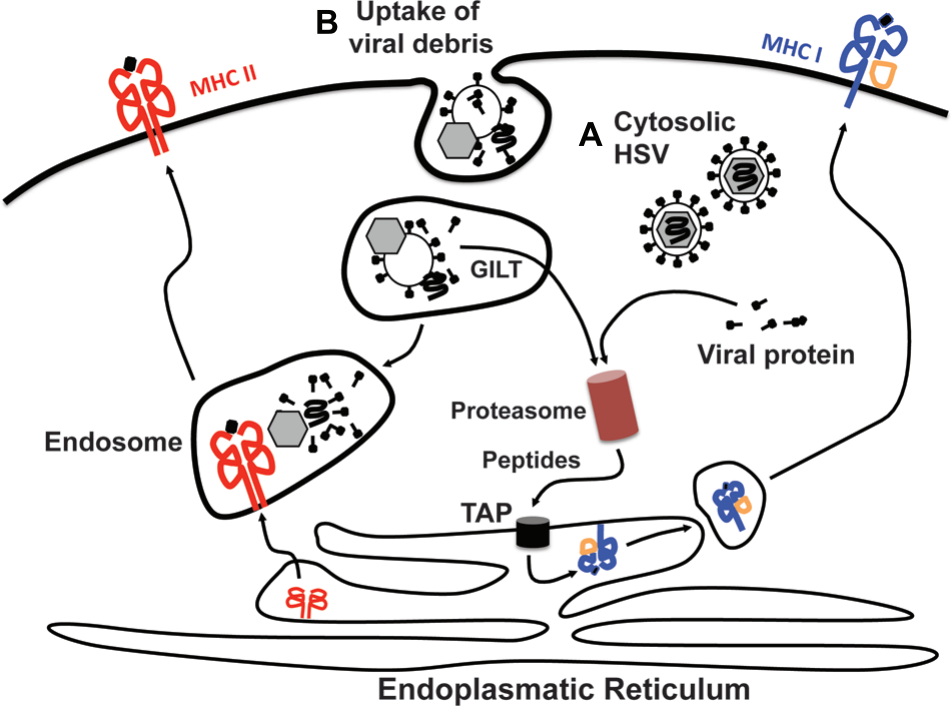
**Access of viral antigens to MHC class I and MHC class II antigen presentation pathways.** There are two principle sources from which viral antigens can be delivered into the antigen presentation machineries. Viral antigens can be derived either from within the cytosol of productively infected cells **(A)** or via ingestion of viral debris or virus-infected cells **(B)**. The presentation of cytosolic antigens on MHC class I molecules requires proteasomal degradation and subsequent transport of the generated peptides into the endoplasmatic reticulum (ER) via the transporter associated with antigen processing (TAP). In the ER, peptides are loaded onto MHC class I molecules and trafficked in vesicles to the cell surface via the Golgi apparatus. This pathway is typically referred to as “direct presentation.” Peptides for loading onto MHC class I molecules can also be generated from exogenous proteins that have been engulfed by the cell in a process termed “cross-presentation.” For this to occur the exogenous proteins contained within endosomes need to be unfolded, which likely involves gamma-interferon-inducible lysosomal thiol reductase (GILT), before being translocated into the cytosol. Once in the cytosol they can be fed into the MHC class I presentation pathway as outlined for cytosolic antigens. The endosomal content also provides the major source for antigens that are loaded onto MHC class II molecules. It should be noted, however, that this is a very simplified view of the processes underlying cross-presentation. More detailed descriptions can be found elsewhere (Joffre et al., 2012; Nair-Gupta and Blander, 2013).

A central role of DCs in viral infections is to activate naïve antigen-specific CTLs in the lymph node. Following the conversion of naïve CTLs into effector cells, these migrate into the tissues, where they actively contribute to reducing the number of infected cells, such as keratinocytes. Several studies have demonstrated that DCs isolated from the lymph nodes draining the site of HSV infection can efficiently stimulate virus-specific T cell responses (Zhao et al., 2003; Allan et al., 2006; Bedoui et al., 2009b). Analysis of the different DC subtypes present in murine lymph nodes following epidermal HSV infection revealed that early presentation of HSV-derived, MHC class I-restricted antigens was restricted to lymph node-resident CD8^+^ DCs (Allan et al., 2003) and even its CD24^+^ CD8^-^ precursors (Bedoui et al., 2009a). Together with comparable results obtained in a model of vaginal HSV infection (Zhao et al., 2003), these findings challenged the prevailing view at the time that skin-derived DCs would not only collect antigens in the skin and transport it to the lymph node, but were also responsible for presenting it to naïve CTLs (Carbone et al., 2004). A subsequent study revealed that pharmacological blockade of DC migration from skin to the lymph node impaired CTL priming in response to HSV skin infection (Allan et al., 2006). This indicated that although lymph node-resident DCs were key to CTL priming, their ability to present HSV-derived antigens on MHC class I molecules still depended on the immigration of migratory DCs from the site of infection. As pharmacological blockade should not affect potential passive drainage of free virions from the skin to the lymph node, these insights also imply that MHC class I presentation by lymph node-resident CD8^+^ DCs was not a consequence of drained virus actively infecting CD8^+^ DCs in the lymph node (**Figure 2**). Consistent with this conclusion, free virions could only be detected in lymphatics after HSV infection via the subcutaneous route, but not following epithelial inoculation (Lee et al., 2009). Thus, rather than CD8^+^ DCs displaying antigens on MHC class I molecules as a result of direct presentation, the suggestion was that antigens collected in the skin by migratory DCs were passed on to CD8^+^ DCs in the lymph node (Carbone et al., 2004). The exact mechanism enabling CD8^+^ DCs to receive antigens from migratory DCs is still enigmatic, but could potentially involve active transfer of antigens between the two cells via exosomes (Thery et al., 2009) or gap junctions (Mazzini et al., 2014). The observation that HSV-infected DCs readily undergo apoptosis also opens up the possibility that CD8^+^ DCs access viral antigens in the lymph node by phagocytosing cellular debris from HSV-infected migratory DCs that have died in the skin or en route to the lymph node. This view is consistent with *in vitro* studies showing that uninfected human Mo DCs can engulf material from apoptotic, HSV-infected Mo DCs and subsequently present the collected antigens to HSV-specific CTLs (Bosnjak et al., 2005). The notion of transfer of antigens from DCs originating from the site of infection to immature DCs residing in the draining lymph node has interesting implications. For example, it likely represents a means through which antigens collected by a single DC at the site of infection can be distributed over multiple lymph node resident CD8^+^ DCs, essentially enabling a greater number of DCs to present the peripherally collected antigens to cognate naïve T cells. Transfer of antigens can also be viewed as a strategy to overcome some of the sophisticated immune evasion strategies that HSV has evolved during its long co-existence with the human host. For example, by ensuring that antigens “escaping” from dying HSV-infected DCs are made available for presentation by uninfected DCs, the capacity of HSV to drive infected DCs into apoptosis becomes biologically less relevant for the induction of protective CTL responses. Similarly, it could be argued that the transfer of antigens counteracts the very effective ability of HSV to interfere with expression and peptide loading of MHC molecules by infected cells (Piguet, 2005).

## CROSS-PRESENTATION OF HSV-DERIVED ANTIGENS BY DCs

The notion that CD8^+^ DCs take up virally infected material, rather than being directly infected, implies that HSV-specific CTL priming relies on DCs shunting exogenous antigens into the MHC class I presentation pathway (**Figure 2**) in a process referred to as cross-presentation (Ackerman and Cresswell, 2004; Kurts et al., 2010; Segura and Villadangos, 2011; Joffre et al., 2012; Nair-Gupta et al., 2014). Support for the relevance of cross-presentation in this context comes from the observation that HSV infection of mice lacking gamma-IFI lysosomal thiol reductase (GILT) results in impaired HSV-specific CTL responses (Singh and Cresswell, 2010). As GILT-deficient DCs can only present HSV-derived antigens to CTLs when directly infected with HSV, but not upon exposure to HSV-infected bystander cells, defective CTL priming in GILT-deficient mice is consistent with a selective defect in cross-presentation (Singh and Cresswell, 2010). The ability of CD8^+^ DCs to cross-present HSV-derived antigens was further shown *in vitro* by exposing uninfected CD8^+^ DCs to bystander cells that were infected with gH- or gB-deficient HSV mutants (Jirmo et al., 2009). The gH/gB-deficiency incapacitated the virus from forming infectious virions, ensuring that productive infection only occurred in the bystander cells, but not the CD8^+^ DCs. As the bystander cells were chosen so they themselves could not present the antigens, the proliferation of HSV-specific CTLs observed in these *in vitro* experiments could therefore be attributed to CD8^+^ DCs cross-presenting antigens that they had engulfed from the infected bystander cells. These findings together with impaired HSV-specific CTL priming in mice lacking CD8^+^ DCs due to the absence of the transcription factor basic leucine zipper transcription factor, ATF-like 3 (batf3; Nopora et al., 2012) emphasize that cross-presentation could be an important means by which CD8^+^ DCs stimulate HSV-specific CTL responses.

Interestingly, uptake of debris from dying or dead, HSV-infected cells not only serves to deliver antigens, but also appears to provide crucial innate signals to the CD8^+^ DCs. Following HSV skin infection, the ability of CD8^+^ DCs to propagate effective CTL priming was shown to depend on their expression of TLR3 and TIR-domain-containing adapter-inducing IFN-β (TRIF), while other TLRs that signal through MyD88 were dispensable (Davey et al., 2010). Considering the endosomal location of TLR3 (Schulz et al., 2005), and the fact that HSV-encoded viral RNA intermediates are abundant in HSV-infected cells (Weber et al., 2006), it appears likely that the TLR3 pathway is engaged in CD8^+^ DCs as they engulf debris from HSV-infected cells. How TLR3-mediated stimulation in CD8^+^ DCs subsequently contributes to more efficient HSV-specific CTL priming remains to be resolved. However, in this context it is noteworthy that *in vitro* equivalents of mouse CD8^+^ DCs secrete IFN-λ in response to HSV-1 exposure and that poly(I:C)-induced IFN-λ production depends on the presence of TLR3 (Lauterbach et al., 2010). It is therefore possible that IFN-λ might be an important downstream element of the TLR3 response that enables CD8^+^ DCs to prime HSV-specific CTL responses with optimal efficiency.

**FIGURE 3:**
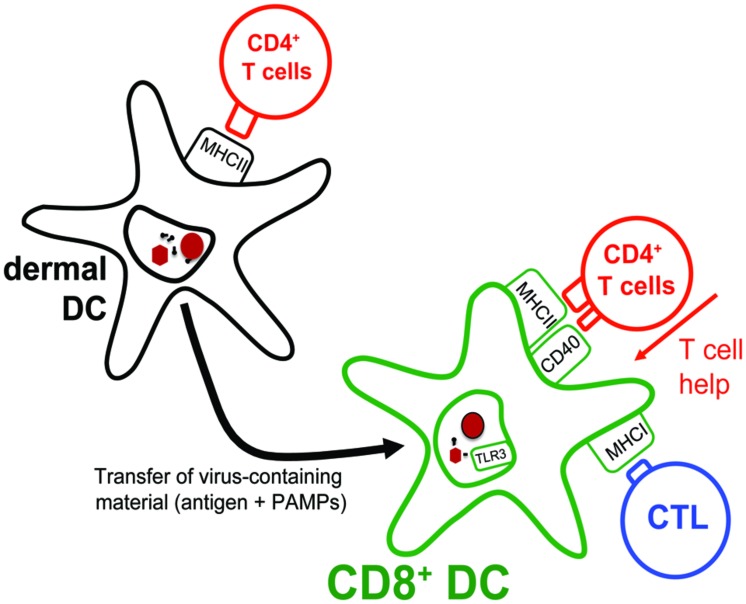
**Transfer of antigens from migratory DCs to lymph node-resident CD8^+^ DCs.** Dermal DCs, arriving in the lymph node draining the site of HSV skin infection, present antigens to CD4^+^ T cells. Interestingly, dermal DCs themselves are inefficient at presenting the collected antigens to HSV-specific CTLs. Instead, they transfer their antigenic cargo to lymph node-resident CD8^+^ DCs that subsequently present these to both HSV-specific CD4^+^ T cells and CTLs. Notably, by optimizing the capacity of CD8^+^ DCs to present HSV-derived antigens on MHC class I molecules, the CD4^+^ T cells also assist in augmenting the overall CTL response to the infection.

Thus, there are several lines of evidence in support of the view that CD8^+^ DCs gain access to viral antigens from exogenous sources rather than as a consequence of being productively infected (**Figures 2** and **3**). However, definitive proof that cross-presentation is required for HSV-specific CTL priming is missing and the relative contribution of direct and cross-presentation to the overall magnitude of anti-viral T cell immunity still remains to be unraveled.

## MHC CLASS II-RESTRICTED PRESENTATION OF HSV ANTIGENS BY DCs

It is thus plausible that CD8^+^ DCs receive HSV antigens from migratory DCs in the lymph node. However, reducing the role of migratory DCs during HSV skin infections to that of simple providers of antigens for lymph node-resident DCs is a gross oversimplification. In fact, viable dermal DCs isolated from the lymph nodes draining the site of HSV infection have been demonstrated to stimulate the differentiation of naïve HSV-specific CD4^+^ T cells into effector cells (Bedoui et al., 2009b; Lee et al., 2009). These HSV-specific CD4^+^ T cell responses are not only important for the resolution of the virus infection in the skin (Gebhardt et al., 2011), but intriguingly also contribute to the efficiency with which CTLs are primed in a process referred to as “T cell help” (Bevan, 2004). This has been documented by studies showing that the magnitude of the primary HSV-specific CTL response following subcutaneous HSV infection is impaired in the absence of CD4^+^ T cells (Smith et al., 2004; Rajasagi et al., 2009). Mixed bone marrow chimeras revealed that the very same DCs that stimulated the HSV-specific CTL response *in vivo* also needed to receive signals from cognate CD4^+^ T cells. With CD8^+^ DCs being the only DC subset capable of presenting HSV-derived antigens to CTLs early in the response and CD8^+^ DCs also displaying HSV-derived antigens on MHC class II molecules *ex vivo* (Bedoui et al., 2009b), the likely scenario is that it is the CD8^+^ DCs that require T cell help to efficiently prime the CTL response (**Figure 3**). However, how exactly CD4^+^ T cells improve the capacity of CD8^+^ DCs to drive the differentiation of the few naïve HSV-specific CTLs into fully armed effector cells needs to be examined in more detail.

Thus, while some migratory DCs, potentially those that are infected by HSV, can serve as a source of antigens for other DCs (Carbone et al., 2004), there are also skin-derived DCs in the lymph node that have likely obtained HSV-derived antigens without being productively infected. It is possible that uninfected dermal DCs might take up HSV-derived antigens in a similar manner as lymph node-resident DCs, namely by engulfing debris from dead, infected migratory DCs that have drained into the lymph node. However, this appears unlikely as skin-derived DCs mature during their migration from the tissues to the lymph node and most evidence indicates that mature DCs have rather poor abilities to take up new antigens (Reis e Sousa, 2006). What seems more likely is that dermal DCs collect antigens in the skin from infected keratinocytes or other infected epidermal cells, such as γ/δ-T cells or even other DCs (Puttur et al., 2010). To account for potential issues related to the basal membrane at the dermo-epidermal junction physically separating dermal DCs from infected epidermal cells, it has been suggested that dermal DCs might obtain antigens from HSV-infected LCs that die as they transit through the dermis en route to the lymph node (Cunningham et al., 2010). Whether this is the dominant way through which dermal DCs obtain viral antigens in the skin, or whether dermal DCs might be directly exposed to dead keratinocytes within lytic lesions that have eroded the dermo-epidermal barrier needs to be investigated. It is also conceivable that dermal DCs could send protrusions into the epidermis as has been suggested to occur in the intestine (Rescigno et al., 2001). Any examination of this interesting question should furthermore take into account that some dermal DCs have been observed in very close proximity to hair follicles where HSV replicates extensively (Bursch et al., 2007; Eidsmo et al., 2009) and, that as the lesion size gets larger over time additional skin-derived DC subsets, such as LCs and CD103^+^ DCs, also start presenting HSV-derived antigens to CD4^+^ T cells in the draining lymph node (Bedoui et al., 2009b). Intriguingly, in whatever way these migratory DCs gain access to HSV antigens in the later stages of the infection, CD103^+^ DCs can also feed the acquired antigens into the MHC class I pathway (Bedoui et al., 2009b). The increasing appreciation that lymph node-resident CD8^+^ DCs and migratory CD103^+^ DCs are very closely related (Crozat et al., 2010b; Bachem et al., 2012) helps explain why amongst the migratory DCs only the CD103^+^ DCs gain the capacity to stimulate naïve HSV-specific CTLs later in the response. It remains puzzling, however, why the CD103^+^ DCs do not participate in the early phase of HSV-specific CTL priming. Their late contribution could be related to providing a second antigenic hit to expanding CTLs before they leave the lymph node. It is also possible that CD103^+^ DCs regulate tissue-homing capacities of the responding CTLs, as has been documented in the intestinal immune system (Stock et al., 2013). Clearly more research is required to delineate the processes through which skin-derived and lymph node-resident DCs obtain HSV antigens and what the consequence of their antigen presentation is.

## ANTIGEN PRESENTATION BY DCs TO ANTIGEN-EXPERIENCED T CELLS IN INFECTED TISSUES

The above has focused on the role of DCs in the differentiation of naïve T cells into HSV-specific effector T cells that subsequently depart the lymphoid tissues and enter infected sites. Accumulating evidence suggests that DCs also play an important role in assisting activated T cells to exert effector functions within primary infected tissues (Bedoui and Gebhardt, 2011). For example, locally differentiated Mo DCs were recently shown to stimulate IFN-γ secretion by effector CD4^+^ T cells infiltrating mucosal tissues of vaginally infected mice (Iijima et al., 2011). A more recent analysis into the contribution of DCs to local CD4^+^ T cell and CTL responses following HSV skin infection, made the observation that CD4^+^ T cells and CTLs differed substantially in their requirement for DCs to elicit local effector function (Macleod et al., 2014). While HSV-specific CD4^+^ T cells secreted IFN-γ upon recognizing cognate antigen on DCs regardless of whether these were infected with HSV or not, IFN-γ secretion by HSV-specific CTLs was only elicited by infected cells. The latter were predominantly keratinocytes, but also included DCs and γ/δ-T cells. It will be exciting to examine the mechanistic basis of why only infected DCs prompted effector function by HSV-specific CTLs, while DCs that presented HSV-derived antigens to naïve CTLs in the lymph node showed no signs of being infected were protected. How this relates to reports suggesting that elimination of DCs by effector T cells represents a negative feedback regulation on T cell priming (Wong and Pamer, 2003; Yang et al., 2006) should also be investigated by future work.

## CONCLUDING REMARKS

By sensing HSV through pattern recognition receptors, collecting and delivering HSV-derived antigens to the lymph node, stimulating naïve HSV-specific T cell responses and contributing at sites of infection to efficient effector functions by activated T cells, DCs take center stage in the coordinated innate and adaptive immune responses to primary HSV infections. Despite our growing understanding of the intricate interactions between DCs and HSV that not only impact on immunity to the primary infection, but also appear to be of importance in latency (St Leger and Hendricks, 2011), our abilities to prevent new infections or to eliminate the virus from infected individuals are still disappointingly limited (Johnston et al., 2011). Considering the global disease burden associated with HSV infection and the distinct potential for severe complications, such as vertically transmitted neonatal HSV infections, HSV encephalitis or HSV keratitis, more research is necessary to fully understand how the immune system responds to HSV infection.

## Conflict of Interest Statement

The authors declare that the research was conducted in the absence of any commercial or financial relationships that could be construed as a potential conflict of interest.
